# Applying Sparse Machine Learning Methods to Twitter: Analysis of the 2012 Change in Pap Smear Guidelines. A Sequential Mixed-Methods Study

**DOI:** 10.2196/publichealth.5308

**Published:** 2016-06-10

**Authors:** Courtney Rees Lyles, Andrew Godbehere, Gem Le, Laurent El Ghaoui, Urmimala Sarkar

**Affiliations:** ^1^ Center for Vulnerable Populations & Division of General Internal Medicine at the Zuckerberg San Francisco General Hospital Department of Medicine University of California San Francisco San Francisco, CA United States; ^2^ Department of Electrical Engineering and Computer Science University of California Berkeley Berkeley, CA United States

**Keywords:** Twitter, machine learning, social media, cervical cancer, qualitative research

## Abstract

**Background:**

It is difficult to synthesize the vast amount of textual data available from social media websites. Capturing real-world discussions via social media could provide insights into individuals’ opinions and the decision-making process.

**Objective:**

We conducted a sequential mixed methods study to determine the utility of sparse machine learning techniques in summarizing Twitter dialogues. We chose a narrowly defined topic for this approach: cervical cancer discussions over a 6-month time period surrounding a change in Pap smear screening guidelines.

**Methods:**

We applied statistical methodologies, known as sparse machine learning algorithms, to summarize Twitter messages about cervical cancer before and after the 2012 change in Pap smear screening guidelines by the US Preventive Services Task Force (USPSTF). All messages containing the search terms “cervical cancer,” “Pap smear,” and “Pap test” were analyzed during: (1) January 1–March 13, 2012, and (2) March 14–June 30, 2012. Topic modeling was used to discern the most common topics from each time period, and determine the singular value criterion for each topic. The results were then qualitatively coded from top 10 relevant topics to determine the efficiency of clustering method in grouping distinct ideas, and how the discussion differed before vs. after the change in guidelines .

**Results:**

This machine learning method was effective in grouping the relevant discussion topics about cervical cancer during the respective time periods (~20% overall irrelevant content in both time periods). Qualitative analysis determined that a significant portion of the top discussion topics in the second time period directly reflected the USPSTF guideline change (eg, “New Screening Guidelines for Cervical Cancer”), and many topics in both time periods were addressing basic screening promotion and education (eg, “It is Cervical Cancer Awareness Month! Click the link to see where you can receive a free or low cost Pap test.”)

**Conclusions:**

It was demonstrated that machine learning tools can be useful in cervical cancer prevention and screening discussions on Twitter. This method allowed us to prove that there is publicly available significant information about cervical cancer screening on social media sites. Moreover, we observed a direct impact of the guideline change within the Twitter messages.

## Introduction

Social networking websites have fundamentally changed the way in which individuals and organizations communicate with each other [1,2]. Almost 75% of adults on the Internet use online social networking sites, including Facebook and Twitter [3], which provide an opportunity for social interaction and a space to share ideas, opinions, and information [1]. Thus, the content shared ranges from news to personal experiences [4], with health-related information commonly shared and discussed. For example, in a recent survey of adults, a large majority (80%) of younger Americans reported that they would share their personal health information on social media sites [5]. Similarly, in our previous qualitative examination of a sample of Twitter messages about mammograms and Pap smears, we found that a substantial proportion of the top messages within a 1-month period were related to personal experiences within these cancer screenings [6]. Despite this, it is unclear whether the types of health communication on social media sites like Twitter contain reliable information or clear health promotion and/or improvement messages in the midst of millions of comments and discussions.

The immense volume of messages on social media sites (eg, more than 500 million Twitter messages sent every day) [7], preclude the use of traditional qualitative methods to analyze most of this text. Therefore, there is a need to apply new methodologies from computer science and statistics to examine this publicly available content. The so-called sparse machine learning techniques offer a way to examine and summarize large amounts of textual data [8], and therefore would be particularly insightful in studying the online social media content related to cancer prevention, screening, and treatment. This methodology is currently used to analyze such content for businesses (eg, to generate advertising that is tailored to specific online discussion topics) [9], but it is yet to be implemented in biomedical and cancer research.

For this paper, we sought to explore the feasibility of these machine learning approaches to analyze Twitter content about cervical cancer and Pap smears. Specifically, we examined Twitter messages about these topics before and after the US Preventive Services Task Force (USPSTF) guideline change on March 15, 2012 [10] (Table 1), using a sequential mixed method approach.

**Table 1:** Summary of changes in the USPSTF guidelines for Pap smear screening.

	Guideline prior to 2012	Updated guideline release in March 2012
Frequency of screening	At least every 3 years	Every 3 years
Age to begin screening	Within 3 years of initiating sexual activity, or age 21	Age 21
Human papillomavirus (HPV) testing	Insufficient evidence to make a recommendation	No HPV screening for those under age 30

## Methods

### Data Source

For this study, we gathered and stored messages from a specific online social networking site, Twitter, the second largest social networking site after Facebook.[11] Twitter allows individuals to share information in short text messages called “tweets” that are 140 characters or less. Twitter is largely a public forum where users follow real-time information, and accounts range from personal (from friends and family to celebrities and politicians) to organizational (including news sources, national associations, and local groups). Using an application programming interface from a third-party Twitter platform (Topsy©), we were able to access a large collection of more than 20,000 Twitter messages originating in the US about Pap smears and cervical cancer over a 3-year time period (ie, 2009-2012). We restricted our data collection to messages citing our cancer-specific query keywords (“Pap smear,” “Pap test”, or “cervical cancer”, including common misspellings).

Within the raw dataset, we calculated the raw frequency data for messages related to cervical cancer screening during an entire 6-month time period, that is, we examined the total number of occurrences of the keywords on Twitter from January 2012 to June 2012. We then divided the data into two distinct time periods: before (January 1, 2012—March 13, 2012) and during or after (March 14, 2012—June 30, 2012) the USPSTF guideline change.

### Mixed Methods Analysis

As we wanted to establish the face validity of using newer machine learning methods to analyze Twitter data, we used a sequential mixed methods approach for our study by first completing a quantitative summarization of the data using sparse principal component analysis, followed by qualitative content analysis of example messages.

#### Sparse Principal Component Analysis

After downloading the data, we first used an exploratory quantitative analysis using machine learning algorithms. Machine learning encompasses a set of statistical and computational tools, which assist in extracting meaning and insight from very large collections of data (see Multimedia Appendix 1 for more information). We used “sparse’’ machine learning algorithms [12,13] as an alternative to extremely large volumes of keywords in the machine learning classification process (as in Latent Semantic Indexing) . The sparse algorithms attribute a zero weight to as many keywords as possible, limiting the result to a short list of keywords with strong interrelationships and the most representative messages using those keywords. Therefore, in practice, sparsity enables interpretability by an analyst [8,14].

Specifically, we used a linear algebraic factor-model approach called sparse principal component analysis (SPCA), which split the results into a number of distinct topics. Each topic was characterized by a short list of keywords that were all determined to be correlated. In addition, few Twitter messages that were most strongly and statistically associated with the topic were identified. In practice, SPCA was used both as a topic modeling algorithm (while considering the list of keywords) and clustering algorithm (while considering the example Twitter messages). This form of SPCA was used in our previous studies [14-16].

In order to apply the SPCA approach to Twitter data (as opposed to other forms of text data, like full news articles), we modified the list of stop-words in our algorithm. For example, the text attached to the symbol “@” from the result terms was removed as it represents a username on Twitter rather than a part of the message content itself, and the uniform resource locator (URL) addresses were also removed since they were not considered as intelligible text. Hashtags (“#”) were permitted as they allowed users to indicate that their messages were related to the same topic (eg, #cervicalcancer) but did not change the meaning of the tweet. We restricted our analysis to messages tagged as “English” in the main dataset (but we handled the remaining non-English messages as described in more depth below). Finally, we allowed retweeted (indicated by “RT”) messages within our dataset and only the original message as an individual tweet that could be categorized in the cluster analysis was included, but that message was weighted more heavily in the results based on its appearance in the dataset.

As an output of our analysis, the SPCA algorithm required fixed parameters such as number of topics, terms per topic, and tweets per topic for the results. Based on our previous work [8], we specified five keywords per topic, and we allowed the program to produce the total number of topics on the entire datasets. We compared the results for different models using 10, 15, and 20 tweets per topic to identify the one that explained the highest variation in the discussion topics. We used the cumulative Frobenius norm to find out which model explained the highest proportion of the variance with the fewest number of topics, to assess the best performing model [17].

On identifying the best model, we recorded the singular value criterion, for which higher values indicate a higher prevalence of the topic within the entire set of messages (ie, more messages similar to that topic within the entire dataset) and the five keywords most strongly associated with each topic, which were automatically generated from the algorithm. As we used a sparse approximation of principal component analysis, there was a small degree of shuffling in the topic order by singular value criterion.

#### Qualitative Analysis of Example Tweets

We used the example tweets and keywords from the quantitative machine learning SPCA to conduct a qualitative review of the output. In particular, we read all example tweets from the top 10 relevant topics in both the baseline and follow-up periods to determine the nature and cohesiveness of the topics. By use of open coding [18,19] we determined: (1) the primary discussion category of each topic, and (2) the percentage of tweets within that topic that were unrelated to the main message (noting the general content area of the off-topic message). Three members of the research team (CRL, GL, and US) independently read all the tweets to categorize the topics, informed by our previous qualitative methods in a similar dataset [6]. For example, we used broad categories of “health promotion” and “personal experiences with screening” to start the coding process, but allowed new categories to emerge as we read through all tweets independently. The coding team discussed the categorization of topics to generate consensus about the primary meaning of the messages in each topic, focusing mostly on the topic labels in which there was some baseline coder differences in categorization. The coding team also determined the final percentages of messages that pertained to the overall topic category (ie, the cohesiveness of the messages) of the topic. Finally, we selected a single example tweet that represented each topic category. The US member of the research team made the final decision on the categorization and cohesiveness of the topics in case of any disagreement within the coding team.

In a few cases, the results included non-English topics despite our attempt to limit the sample to English messages only. This is most likely because the “English” designation within the Twitter data is imperfect for some messages. Non-English messages most often involved the word “Pap” with a different meaning in another language—these were reported below but not analyzed in the qualitative phase of the study. In addition, some topics returned a smaller number of tweets than specified, largely due to the amount of retweeting of an identical message that was weighted more in the output.

## Results

**Figure 1:**
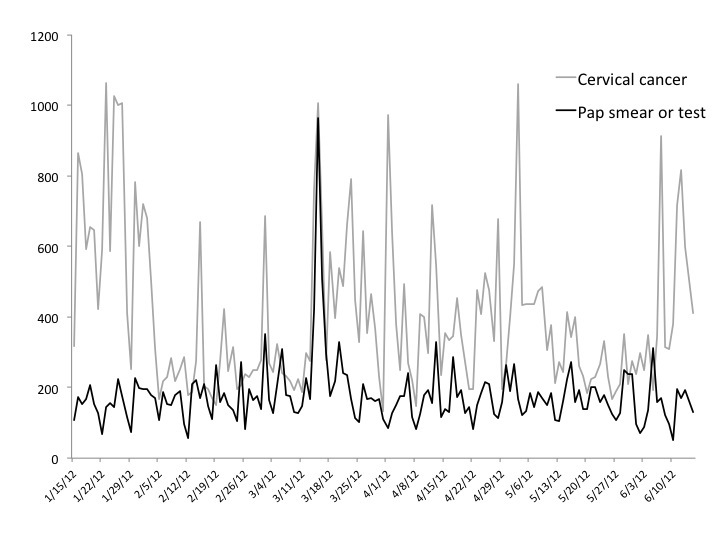
Total mentions of January–June 2012.

Figure 1 displays the total mentions or raw count data for the terms “cervical cancer” and “Pap smear” or “Pap test” in the 6 months surrounding the guideline changes. Immediately following the new USPSTF 2012 guidelines announcement in mid-March, there was an increase in messages containing “Pap smear” or “Pap test”: starting from around 300 mentions in the previous month and spiking to 1000 mentions when the announcement was released. In contrast, the search term “cervical cancer” fluctuated throughout the entire 6-month window.

Next, after dividing the Twitter messages into baseline and follow-up periods surrounding the guideline change, there were 2,549 messages about cervical cancer on Twitter in the baseline period and 4,673 messages in the follow-up period.

**Figure 2:**
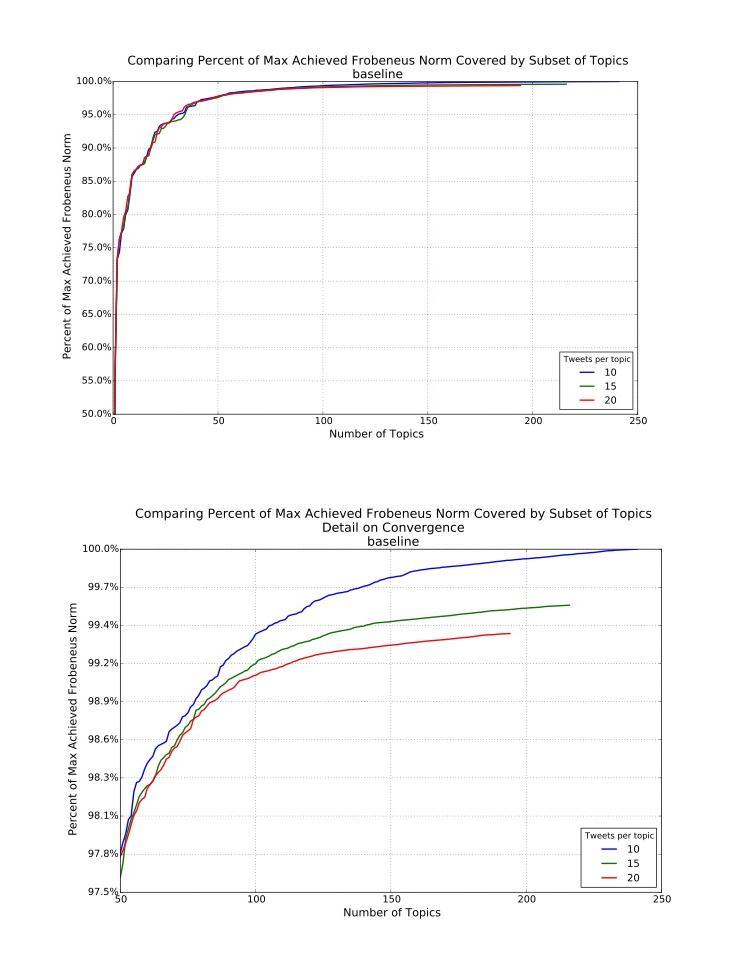
Baseline Frobenius norm plots comparing models specifying 10, 15, and 20 tweets per topic.

**Figure 3:**
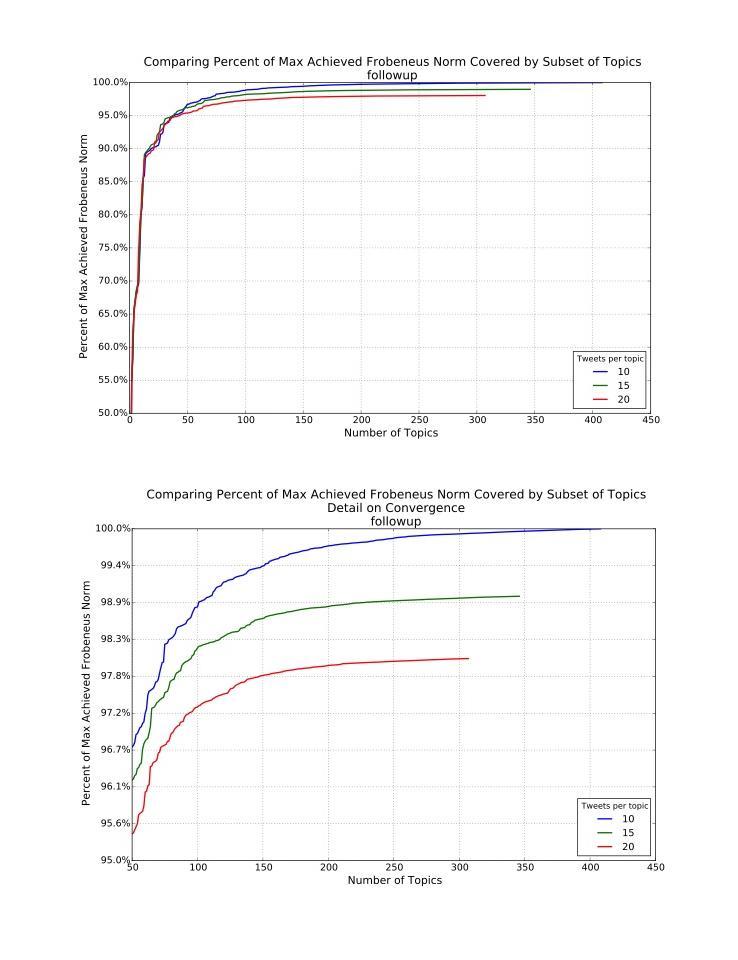
Follow-up Frobenius norm plots comparing models specifying 10, 15, and 20 tweets per topic.

The clustering analysis revealed that the models gave similar results overall, with marginally better performance of 10 tweets per topic as more topics were generated (Figures 2 and 3), that is, overall the 10-tweet model explained the most variance in the discussion topics in the fewest number of topics, but this difference only emerged in explaining the last 10% or so of the variance rather than in the early topic differentiation.

The qualitative analysis of the 10 tweets per topic results are shown in Table 1 (preguideline change) and Table 2 (postguideline change). This analysis showed that about 20% of the content in both time periods was irrelevant, that is, two top topics in both the baseline and follow-up periods were non-English results or nonapplicable messages that used the word “pap” as an abbreviation for another idea.

In terms of the content of the topics that were generated, Table 2 also summarizes the primary category of each topic, the singular value criterion, the proportion of tweets that did not match the overall topic category, the 5 keywords most strongly associated with the topic, an example tweet, and other notes or comments about the topic. The qualitative analysis revealed that the most common topics were about cervical cancer screening promotion or health education.

In the baseline period, the top topic was about screening promotion, followed by information on anti-vaccination and that for individuals with abnormal test results. In these baseline results, 10–40% of the messages were different from the overall topic. In the follow-up period, three of the top topics were directly about the 2012 USPSTF guidelines, with very strong cohesiveness of these topics (ie, very few other messages besides USPSTF guideline information). Several of the message categories in the follow-up period expressed gratitude or relief about not having to have annual Pap smears any longer, which is a direct reflection of the updated 2012 USPSTF guideline stating that women with normal results can now wait 3 years between Pap smears. All the remaining top topics in the follow-up period were decidedly mixed without any single discussion topic dominating the messages.

**Table 2:** Summary of top 10 topics in the baseline and follow-up periods.

Topic	Singular value	Proportion of messages off topic (%)	Example Tweet	Top 5 keywords	Information about “off-topic” tweets
**Baseline period (before guideline change) from January 1–March 13, 2012**					
Non-English	14.10	N/A	Non-English	Non-English	All non-English (Indonesian) tweets with key English words
Screening promotion	11.79	40	Get Tested For Cervical Cancer #cancer #cervicalcancer #paptest #HPV #HPVvaccine #women #health #womenshealth	study, women, #cancer, caught, #papsaveslives	Other 4 messages about cancer survival/cure rate
Education about abnormal test results	6.23	30	Learned in class: women who have an abnormal pap smear result and may have a B12 deficiency should be tested. It can cause a false positive! If you’re under 26, the HPV vaccine can help prevent you from ever having to hear the words “abnormal Pap test results.”	abnormal, doctor, recommended, chances, colposcopy	Other messages: 2 jokes; 1 survival rate
Anti-HPV vaccination information	8.03	40	This Foolish Cancer "Prevention" May Only Extend Your Life by 2.8 Days.	life, foolish, extend, days, #tdh	Other messages: 1 cancer awareness month; 1 education; 1 testimonial; 1 political
Report linking screening to increased survival	5.50	10	Regular Pap Smear Boosts Cervical Cancer Survival: Study: Screening is important even after HPV vaccine, experts say	survival, regular, boosts, health day, including	Other message: education about treatment
Jokes	7.54	30	I got scheduled for a pap smear today. Happy Valentine's Day.” <== At least it's being touched on V-Day	doesn, period, dirty, scheduled, question	Other messages: 2 addressing HPV stigma; 1 education about screening
Education/Screening promotion	5.58	10	We're here, we're queer, pap smear! January is cervical health awareness month, read this: #PublicCervixAnnouncement	health, awareness, care, cdc, remind	Other message giving a personal opinion
Screening promotion with a personal song	8.02	10	Please retweet if you have ever had a Pap Smear (especially if you sang Footloose)	work, retweet, footloose, sang, laser	Other topic was political/unrelated
N/A Irrelevant	7.68	N/A	Smead Slash/Jacket, Letter, 11 Point, Blue, 25 per Pack (75431): 11 pt stock. Holds 8-1/2-inch W x 11-inch H pap...	holds, jacket, stock, letter, inch	Only 2 total tweets for this topic
Education/Screening promotion	5.32	10	It is Cervical Cancer Awareness Month! Click the link to see where you can receive a free or low cost Pap test....	annual, free, month, screening, appt	Other message a joke
Education/Screening Promotion	5.33	0	What are some ways to lower HPV risk: the usual know who you are having sex with, don't have sex, have sex later, get pap smears, std test	smears, told, prevention, kill, relying	Other messages: 5 about survival; 3 general education; 2 promotion
Jokes	4.99	30	All women (and any men who love women--which is all of you in some way!) please read this on Cervical Health Month/HPV This indian man taking my prescription is flirting with me hard. It's unnerving because he looks like that indian man that did my pap smear	man, love, chance, bono, chaz	Messages: 6 jokes (most referencing celebrities); 2 screening promotion; 1 education
**Follow-up period after guideline change: March 14 to June 30, 2012**					
News headlines about guideline change	11.59	0	New Screening Guidelines for Cervical Cancer - Fox News	guidelines, annual, screening, #cnn, corrected	Tweets mostly from news organizations
Non-English (relevant English key words)	14.97	0	Non-English	Non-English	All non-English (Indonesian) tweets with key English words for each tweet in this topic, including “abnormal” “pap smear” “HPV”
Non-English (relevant English keywords)	12.02	0	Non-English	Non-English	All non-English (Indonesian) tweets with key English words for each tweet in this topic, including “abnormal” “pap smear” “HPV”
News headlines on panel recommendations	9.89	20	Health panel: Pap tests needed only every 3 years: NEW YORK (Reuters Health) - Women only need to get a Pap test...	years, panel, health, women, safe	Other messages: 1 educational; 1 political
Health promotion – Female-to-female empowerment messaging	7.94	0	Ladies: Stop putting it off and go have a pap smear. A couple of minutes of discomfort and embarrassment might save your life. Seriously.	ladies, video, learn, defense, routine	All health promotion focused on prevention
Mixed category	7.82	N/A	You know you hate your job when you are excited to leave for two hours for a pap smear.	call, mom, hate, mammogram, dad	Messages: 4 jokes; 3 free resources for testing; 2 guideline change; 1 health promotion; 1 education about screening
Rejoice, no annual pap!	6.35	40	Women Rejoice: Time to Bid Farewell to Your Annual Pap Smear - The Atlantic http://t.co/eIsAegw5 #health	time, rejoice, bid, farewell, atlantic	Other messages: 1 political; 1 joke; 2 education
Mixed category	5.87	N/A	Encourage your mom to get a Pap test this Mother's Day! They're free at 19 PPIN health centers next week! Had The Talk w/ my 6YO: “One day you too shall become a woman & you'll cancel your pap smear to have your hair done.”	woman, free, maintain, order, pbr	Messages: 3 jokes; 4 resources for free Pap smear; 2 promotion for screening; 1 education
Mixed category	10.90	N/A	The Hairpin guide to abnormal pap smears is awesome. Sometimes, we just need facts about our health wrapped in bowties Making sense of your Pap test: #NWHW	rom, companion, pass, guide, citizenship	Only 7 messages in this topic: 2 education; 3 political; 1 opinion about OB-GYN career; 1 irrelevant
Mixed	11.66	N/A	CBC.Beauty Care: Hypnotherapy & Thin Prep Pap Smear	subjects, fundamental, cliffs notes, prep, praxis	Only 3 tweets in this topic: 1 non-English; 1 about Thin Prep Pap Smear; 1 irrelevant
Education about abnormal Pap smear results	8.50	30	If you have abnormal Pap or HPV test results, your doctor will suggest other tests to make a diagnosis. Pls RT	Abnormal, follow, common, procedure, colposcopy	Other messages: 3 education about screening
N/A irrelevant: Pap smears refer to paparazzi, not cervical cancer	11.09	10	Pap Smear: GIVING THE PAPARAZZI A TASTE OF THEIR OWN DISGUSTING MEDICINE #brilliant	paparazzi, tables, cameras, mag, faces	Mostly using “pap” as abbreviation for paparazzi
Mixed category	10.37	N/A	Phrases that a Bama girl never wants to hear: 1) We need to talk. 2) We found something on your pap smear. 3) Auburn just scored. I am answering tweets for this chat RT @XX: Women's #Health Wednesdays 5/2 12-2PM ET. Topic: Pap Test Talk. Join us and use #SCWHW	talk, send, photo, phrases, panic	Messages: 6 jokes; 4 invitations for Tweet Chat about Pap smears

## Discussion

We successfully employed a sequential mixed-methods approach to analyze specific cervical cancer prevention and screening discussions on the online social media site “Twitter”—first using a quantitative topic modeling approach to parse the large text dataset, followed by qualitative analysis of the example Twitter messages that were pulled from the topic modeling.

### Principal Findings

The machine learning methods provide statistically relevant terms, and indexing these to specific tweets was insightful, particularly when there is rich information shared through external links. However, while the resulting keywords from the quantitative approach were insightful, the true meaning of the messages was much more evident when we analyzed the full text examples for additional context. In other words, machine learning topic modeling required contextualization and content expertise, and much more significant adaptations to the algorithm would be needed to be able to replace human interpretation of the messages. Although the machine learning approach could not replace qualitative analysis altogether, it did allow for a more focused qualitative analysis, because it allowed us to prioritize the voluminous content available online.

Our study demonstrates that social media is a platform in which individuals shared direct information about the new 2012 cervical cancer screening guidelines from the USPSTF. In fact, a large proportion of the top topics in the follow-up period were solely dedicated to this shift in screening recommendations. The content of these topics was so distinct from the baseline topics that we feel confident that a machine learning approach can successfully detect such large public changes in dialogue. In addition, the information shared about the change in guidelines was mostly neutral or positive for eliminating the need to have a Pap smear every year. This finding appears to be more positive than other existing literature on women’s perceptions about the new guidelines, which found that a substantial proportion of women expressed concerns about increasing the intervals between screenings [20,21].

Our findings also demonstrated that a large portion of the cervical cancer messages on Twitter were about health promotion and education, beyond discussions about the 2012 screening guidelines. This included new science about the effectiveness of Pap smears and information (personal stories or lists of free screening sites) to encourage women to get Pap smears, which is consistent with our previous qualitative work [6]. We feel that this provides justification for public health and advocacy groups to continue harnessing social media for delivering messages about screening.

### Limitations

Our small exploratory study has limitations. First, we chose one social media site, Twitter, which has a 140-character limit for each post. While Twitter represents a fast-growing platform for Web-based communication [22], Twitter users are likely to differ from the overall US population—as they are more likely to be younger ( aged 18-29 years), from racial or ethnic minority groups (particularly African Americans) and living in urban or suburban areas [11]. In addition, it is not possible to easily automate the process of classifying Twitter messages by sender (such as from individuals vs. organizational accounts), nor we did not verify whether the messages in our analysis were from accounts that were active over a sustained period of time. Second, we are using only publicly available Twitter content—which is likely to represent the majority of content [23] but differs from privately shared messages. Third, we restricted our study to messages originating in the US, because we were interested in a change in national screening guidelines.

Finally, from a methodological standpoint, we faced some limitations in applying a novel approach, such as a few irrelevant sets of messages. While this is expected to a certain extent, future studies will be able to quantify the expected error when using these methods and perhaps additional refinements to minimize such error. The topic modeling and clustering approach also relies on a simple bag-of-words model of text, which ignores word ordering and natural-language semantic information. All analysis is based on patterns of associations between words. In addition, our method only captures the most prevalent associations between words; if a single word has multiple meanings in different contexts, the less prevalent patterns may be overlooked. Finally, we employed a model, which assumes that each message contains a single topic. Other approaches can identify a mixture of topics within a single message, but are computationally expensive [24,25]. Given that Twitter messages are so short, however, a single-topic model of a message appears to capture important and relevant information.

While preliminarily our findings imply that there is an interest in cancer screening discussions among the ever-growing population using online social media. Our work argues for further transdisciplinary study into cancer screening promotion via social media, whether in a peer-to-peer or in an expert-recommendation fashion.

## Abbreviations

SPCA: sparse principal component analysis
USPSTF: US Preventive Services Task Force

## Appendix

Multimedia Appendix 1 Representing text numerically.
