# Supplementary material for: Applying Sparse Machine Learning Methods to Twitter: Analysis of the 2012 Change in Pap Smear Guidelines. A Sequential Mixed-Methods Study
Source: JMIR Public Health Surveill. 2016 Jun 10;2(1):e21. doi: 10.2196/publichealth.5308 (PMC4920957; doi:10.2196/publichealth.5308)
Supplement: Multimedia Appendix 1 [file publichealth_v2i1e21_app1.pdf]

Appendix A: Representing text numerically

The first step in applying (sparse) machine learning methods to text involves representing the documents in a numerical manner through a process called “vectorization.” Here we describe a simple (yet popular) technique based on the so-called bag-of-words model. Assume that we are interested in vectorizing two short documents:

Document 1	Three colors are orange, red, and yellow
Document 2	Three fruits are orange, apple, and banana

First, common English “stop”-words, such as “and”, “or”, “are”, etc are removed, along with punctuation, and each document is assigned a unique ID number.

1	Three colors orange red yellow
2	Three fruits orange apple banana

Then, we construct a lexicon that consists of a list, pairs and triplets, of all the words that appear anywhere in the documents. We assign a unique ID to each of these entries in the lexicon. The order of words in this “bag-of-words” model is irrelevant, and documents are represented as vectors, or an itemized count of each term in the lexicon.,

Table 1: Example vectorization of two documents

	Three	Colors	Orange	Red	Yellow	Fruits	Apple	Banana	Three colors	Colors orange	…
1	1	1	1	1	1	0	0	0	1	1	…
2	1	0	1	0	0	1	1	1	0	0	…

After vectorization, the corpus can be represented as a numerical array, called the term-by-document matrix, and can be processed using the same appraoches used to analyze numerical data. While this representation of text documents discards some information (the order

of words within the document), it has proven nevertheless very useful in automated text processing [8, 11, 12].
